# Breast clinic referrals – should mastalgia be managed in primary care?

**DOI:** 10.1186/1753-6561-7-S1-O7

**Published:** 2013-01-30

**Authors:** Jamal Alamiri, AJ Lowery, S Rajendran, AD Hill

**Affiliations:** 1 Beaumont Hospital, Dublin, Ireland

## Background

Following centralisation of breast cancer services in Ireland, the number of patients attending symptomatic breast units (SBU) has increased significantly. A considerable proportion of patients referred to SBU present with non-suspicious symptoms and fall into a “low-risk” category for breast cancer. It has been proposed that consideration be given to a GP-delivered service for these patients. Aim: To evaluate SBU attendances and correlate with diagnosis to identify a cohort of patients who may be suitable for management in the primary care setting.

## Methods

Data was collected from a prospectively maintained database on patients attending SBU at Beaumont Hospital from January 2011 – May 2012. Reasons for attendance, outcome of triple-assessment and incidence of malignancy were analysed.

## Results

Findings: 6,019 patients underwent triple-assessment at the SBU in this time-period. 1,959 patients were referred with mastalgia, of whom 1,022 (52.2%) reported mastalgia as their only symptom. The incidence of breast cancer in patients presenting with mastalgia alone was 0.8%; all patients diagnosed with breast cancer in this cohort were over 40 years of age. There was no breast cancer diagnosed in patients under 35yrs referred to SBU with mastalgia, and the majority of these patients had a normal breast examination (S2) (82.57%).

## Conclusion

The incidence of breast cancer in patients referred to SBU with mastalgia as an isolated symptom is extremely low. Patients under-35yrs, with mastalgia as an isolated symptom do not require breast imaging and have a sufficiently low risk of breast cancer that they may be suitable for management in the primary-care setting.

